# Interaction of Red Cabbage Extract with Exogenous Antioxidants in ORAC Assay

**DOI:** 10.3390/ijms27041859

**Published:** 2026-02-15

**Authors:** Oskar Sitarz, Grzegorz Bartosz, Izabela Sadowska-Bartosz

**Affiliations:** 1 Laboratory of Analytical Biochemistry, Institute of Food Technology and Nutrition, Faculty of Technology and Life Sciences, University of Rzeszow, 35-601 Rzeszow, Poland; oskars@dokt.ur.edu.pl (O.S.); gbartosz@ur.edu.pl (G.B.); 2 Doctoral School, University of Rzeszow, 35-310 Rzeszow, Poland

**Keywords:** antagonism, antioxidant interaction, ascorbic acid, gallic acid, glutathione, ORAC, red cabbage, TEMPOL, Trolox, synergy

## Abstract

Understanding interactions between antioxidants is crucial since in biological and food matrices, we are dealing with complex multi-component antioxidant systems. This study aimed to quantitatively characterize interactions of antioxidants in anthocyanin-rich aqueous red cabbage extract with several natural (ascorbic acid, gallic acid, and glutathione) and synthetic (Trolox and TEMPOL) antioxidants and to determine their synergistic or antagonistic nature in the ORAC assay. Four parameters derivable from the ORAC assay (extent of fluorescence protection, lag time, fluorescence half-life t_1/2_, and maximum rate of fluorescence decay) were analyzed in terms of the integrated interaction coefficient (IIC), reflecting the dependence of the analyzed values on the concentration of antioxidants and the sample interaction coefficient (SIC) derived from interaction at a single set of concentrations. IIC analysis revealed synergistic interactions of Trolox with the red cabbage extract on the basis of fluorescence protection, lag time, and t_1/2_. Interactions of TEMPOL with the extract were antagonistic as assessed based on all parameters but the lag time. A correlation between the anodic peak and the lag time and t_1/2_ values was observed for the antioxidants studied. The interactions between antioxidants in complex mixtures are important as they affect the measured total antioxidant activity, which, depending on the nature of the interactions, may be lower or higher than the sum of activities of individual components.

## 1. Introduction

Interactions between antioxidants are of considerable interest, since both in organisms and food products, antioxidants are not present individually but form a complex system within which they interact with one another. An insight into the in vivo interactions between antioxidants is difficult. Therefore, interactions between antioxidants are mainly studied in model in vitro systems, enabling studies of pairs or groups of antioxidants. The validity of extrapolations of the results of such studies to in vivo systems of complex food products has been criticized for several reasons. The reaction conditions used in most assays (solvents, pH, temperature, and the absence of a natural matrix and other compounds such as metal ion chelators) differ from the physiological conditions of antioxidant activity. The time frame of the assays is limited, so slow reactions may not be included. Moreover, the oxidants in some antioxidant assays are synthetic radicals not occurring in nature [1,2,3]. Notwithstanding this justified criticism, in vitro studies are a necessary initial step for controlling variables before moving on to in vivo studies. It has been argued that one step to bringing the in vitro assays closer to the in vivo or food systems may consist in the use of biologically relevant oxidants instead of artificial ones. The ORAC assay, crocin bleaching assay, and evaluation of the prevention of lipid peroxidation are examples of such assays, as they are based on the inhibition of oxidation caused by peroxyl or alkoxyl radicals, which are important oxidants in the body and in food oxidation [4,5,6,7,8]. Of these, the ORAC assay is the most suitable for estimating the activities of hydrophilic antioxidants and their interactions at a neutral pH and physiological temperature for the human organism.

The principle of the ORAC assay is the measurement of the protection of fluorescence of an indicator (most commonly, fluorescein) [7,9]. However, it has been suggested that another parameter that can be derived from the ORAC assay, the lag time of the reaction, is determined by the repair of the fluoresceinyl radical, formed as an intermediate in the oxidation of fluorescein, and is dependent on the one-electron redox potential of the antioxidants studied [10,11]. Testing this hypothesis with the set of antioxidants used was the first aim of this study.

In a previous study [12], we examined the interaction of antioxidants present in red cabbage extract, mainly anthocyanins, with several exogenous antioxidants using ABTS^•^ decolorization and FRAP assays, finding assay-specific differences in the type of interaction. The red cabbage extract is rich in anthocyanins [13,14] but also contains other endogenous antioxidants. It is a complex system due to the presence of multiple cyanidin derivatives and other antioxidants, which allows for the evaluation of the “matrix effect” against pure antioxidants. This system is closer to real systems in which antioxidant supplementation affects cells, organisms, and food products that already possess endogenous antioxidants. The second aim of the study was to analyze interactions in the same systems using the ORAC assay and to compare the results with those from previously used assays. This was of interest since ORAC is a HAT-based assay, in contrast to the SET-based FRAP assay and the ABTS^•^ decolorization assay, mainly involving the SET mechanism [7,9].

Like in a previous study, three natural antioxidants (ascorbic acid, gallic acid, and glutathione) and two synthetic antioxidants (Trolox and TEMPOL) were employed. They represent different classes of compounds with different reactivity patterns. Ascorbic acid (vitamin C), a carbohydrate derivative, is the main water-soluble antioxidant vitamin, essential for preventing connective tissue diseases and providing many other beneficial health effects [15,16]. Gallic acid (3,4,5-trihydroxybenzoic acid), a phenolic, is one of the main plant phenolic acids, exerting numerous health effects, and is important in many industrial applications [17,18]. Glutathione, a thiol peptide, is the main intracellular antioxidant in animal cells and is also important in other organisms [19,20]. Trolox is an analog of vitamin E, a convenient and commonly used standard antioxidant due to considerable solubility in aqueous solutions [21,22]. TEMPOL is a stable nitroxyl free radical broadly used as an antioxidant [23,24].

The third aim of the study was to compare various parameters that can be derived from the ORAC assay and discuss their usefulness in the analysis of the interaction between antioxidants. It could be expected that parameters such as the lag time, suggested to depend on the redox potential of antioxidants [10,11], and a related parameter t_1/2_ may bring more specific information than the AUC, which is affected by several variables [7,9].

## 2. Results

**Figure 1:**
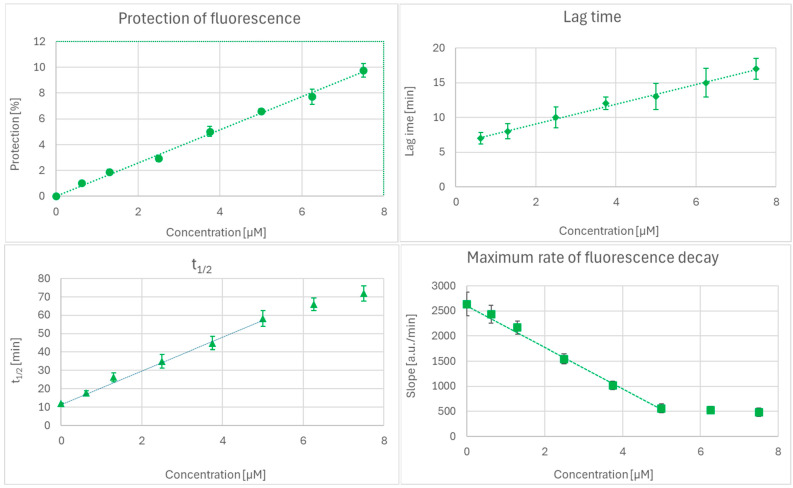
Concentration dependence of the parameters derived from ORAC measurements (extent of fluorescence protection, lag time, t_1/2_, and the maximum rate of fluorescence decay) for Trolox. Mean values ± SD, *n* = 3.

In the ORAC assay, the plots of the extent of fluorescence protection and lag time for pure antioxidants, the red cabbage extract, and combinations of the extract with antioxidants, with respect to the concentration of antioxidants, were linear. t_1/2_ and the maximal rate of fluorescence decay were linear for the lower concentration range of antioxidants (up to about 5 µM), as exemplified by Trolox (Figure 1). In such cases, only this linear range was used for the analysis. The whole range of dependence studied could be fit by a 3rd-order polynomial, but changes in the coefficients were difficult to interpret.

**Figure 2:**
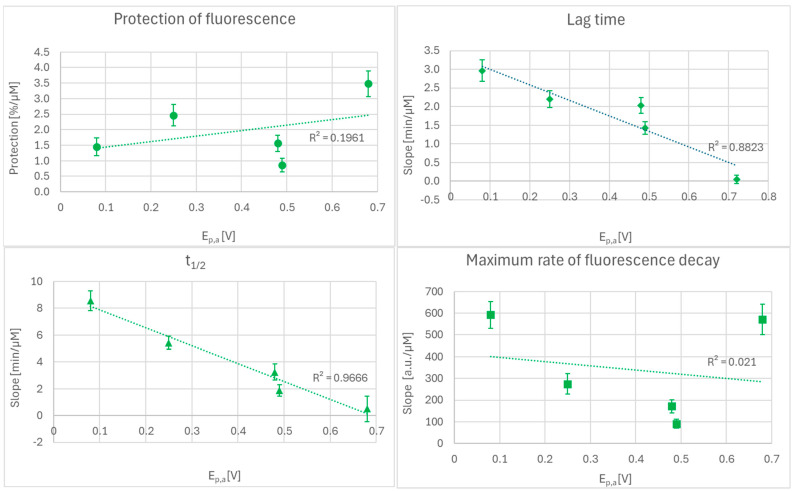
Slopes of the dependence of the extent of fluorescence protection determined from the AUC, lag time, t_1/2_, and maximal slope of fluorescence decrease on the antioxidant concentration studied. E_p,a_, anodic peak potential. Mean values ± SD; *n* = 3.

The slopes for the dependence of lag time and t_1/2_ on the antioxidant concentration were dependent on the E_p,a_ of the antioxidants studied (Figure 2). Despite the low number of cases, the correlation coefficients of the slopes for lag time and t_1/2_ with E_p.a_ of the antioxidants were statistically significant (*p* < 0.01 and *p* < 0.05, respectively). In contrast, there was no significant correlation between the slopes of the concentration dependence of the extent of fluorescence protection and the maximum rate of fluorescence decay on the E_p,a_ of the antioxidants. The lack of dependence of these parameters on the E_p,a_ of antioxidants is understandable as they integrate multiple chemical processes and not just the thermodynamics of a single-electron reaction.

The interaction between the red cabbage extract and the selected antioxidants was evaluated on the basis of all four parameters derived from the ORAC assay by means of the sample interaction coefficients and integrated interaction coefficients.

The sample interaction coefficient (SIC) was calculated for individual samples:

SIC = X_EA_/(X_E_ + X_A_),

where X_E_ is a parameter (extent of protection, lag time, t_1/2_ or the maximum rate of fluorescence decay) measured in a sample containing the red cabbage extract, X_A_ is the same parameter measured in a sample containing an antioxidant, and X_EA_ is this parameter measured in a sample containing both the extract and the antioxidant (at the same concentrations as those used for the determination of X_E_ and X_A_).

The integrated interaction coefficient (IIC) was calculated from the concentration dependence of X_E_, X_A_ and X_EA_:

IIC = (slope of the concentration dependence of X_EA_)/[(slope of the concentration dependence of X_E_) + (slope of the concentration dependence of X_A_)]

The IIC values for the red cabbage extract and the selected antioxidants estimated from the extent of fluorescence protection, derived from the AUC, are shown in Table 1. They were >1 for all antioxidants studied except for TEMPOL. However, a statistically significant positive deviation from the value of 1, indicating a synergic interaction with the red cabbage extract, was obtained only for Trolox, so the interactions of ascorbic acid, gallic acid, and glutathione with the extract should be regarded as additive. A negative deviation from additivity was found for TEMPOL, indicating an antagonistic interaction. No statistically significant differences in IIC were found when comparing values obtained for different concentration ranges of individual antioxidants.

IIC values obtained from the concentration dependence of the lag time demonstrated additive interactions for ascorbic acid, gallic acid, glutathione, and TEMPOL but a synergistic interaction for Trolox (Table 2). No significant differences in IIC were found when comparing values obtained for different concentration ranges of individual antioxidants.

IIC values found for t_1/2_ were >1 for all antioxidants but TEMPOL. Statistically significant differences from the value of 1 (additive interaction) were obtained only for TEMPOL, where the interaction was classified as antagonistic. No significant differences in IIC were found when comparing values obtained for different concentration ranges of individual antioxidants (Table 3).

IICs calculated based on the maximal slope of the fluorescence decay were <1 for all antioxidants. However, statistically significant differences from the value of 1 (antagonistic interaction) were found only for TEMPOL. No significant differences in IIC were found when comparing values obtained for different concentration ranges of individual antioxidants (Table 4).

Average SIC values obtained for the interaction of selected antioxidants with antioxidants of the red cabbage extract, estimated by various types of analysis of ORAC data and calculated based on data presented in Tables S1–S4 are presented in Table 5. The evaluation of the type of interaction based on these values seems less reliable than the use of the IIC. The IIC is a more robust parameter than the SIC, since, by being based on slopes, it eliminates the variability inherent in measurements at individual concentrations. Statistically significant differences from the value of 1 (additive interaction) were obtained only for one concentration range of antioxidants and not the other (interaction of the extract with ascorbic acid, GSH, and Trolox evaluated by fluorescence protection, and interaction of the extract with gallic acid and TEMPOL as evaluated by the lag time). In the case of the average SIC derived from the lag time, the difference between values derived from the two concentration ranges of gallic acid was statistically significant (Table 5). There were significant disparities between the IIC values for t_1/2_ and maximum rate of fluorescence decay (Table 3 and Table 4) and the average SIC values for these parameters. SIC values indicated antagonistic interactions of all antioxidants and did not show synergic interactions of Trolox and additive interactions of ascorbic acid, gallic acid and glutathione with antioxidants from the red cabbage extract, evident from the IIC values for t_1/2_, or additive interactions of ascorbic acid, gallic acid, glutathione and Trolox with the extract, evident from the IIC values for the maximal rate of fluorescence decay.

An even higher variability was observed between individual SIC values, where synergistic or additive (or antagonistic or additive) interaction could be concluded on the basis of results obtained for various concentrations of antioxidants (Tables S1–S4).

## 3. Discussion

The ORAC assay is based on the protection of fluorescein, by antioxidants present in the examined sample, from oxidation by AAPH-derived radicals. It is generally believed that the main species responsible for the oxidation of fluorescein (or another indicator) is peroxyl radicals (ROO^•^) generated from AAPH [5,7,9], although it was suggested based on kinetic analysis that in the absence of antioxidants, alkoxyl radicals formed from peroxyl radicals predominantly oxidize fluorescein to the fluoresceinyl free radical [10,11]. Various parameters that can be derived from AAPH measurements may reflect various facets of these reactions.

The lag time was postulated to reflect mainly the repair of the fluoresceinyl radical by antioxidants rather than the scavenging of the peroxyl radicals [10,11]. The parameter most widely used to analyze the results of the ORAC assay is the “area under the curve” or sum of individual fluorescence values obtained during the kinetic measurement of the decay of fluorescein fluorescence (AUC). The AUC is affected by all reactions occurring in the system (scavenging of peroxyl and alkoxyl radicals and repair of fluoresceinyl radicals). Another parameter derived from the kinetic plots of the fluorescence decay is the lag time, easily visible for some antioxidants and less distinct for other compounds. We also used two other parameters that could be easily derived from the plots of fluorescein fluorescence vs. time of reaction: t_1/2_ and the maximal slope of fluorescence decay. t_1/2_ is mainly related to the lag time, although it can be affected to some extent by the terminal rate of fluorescence decay. This parameter can serve as an objective and automatable surrogate for lag time, eliminating the subjectivity associated with its manual determination. The maximum rate of fluorescence decay depends on the ratio of (sum of reaction rates of remaining antioxidants with the oxidizing radicals + sum of repair of fluoresceinyl radicals by remaining antioxidants) to the rate of reaction of oxidizing radicals with fluorescein.

It has been proposed that the lag time is more pronounced the lower the one-electron redox potential of an antioxidant is compared with that of the fluorescein [10,11]. Results obtained in this study strongly support this view. The anodic peak potential (E_p,a_) for fluorescein is 0.71 V vs. Ag/AgCl electrode [25] while the values for Trolox, GSH, gallic acid, ascorbic acid and TEMPOL are 0.08 V [26], 0.25 V [27], 0.48 V [28], 0.49 V [29], and 0.68 V [30], respectively. Lower redox potential values favor radical repair during the lag phase. The reactions of antioxidants with the peroxyl radical are expected not to depend on their one-electron redox potentials, as all of them are much lower than that of the peroxyl radicals (ca. 1 V) [31].

Based on our earlier experience, we prefer to characterize antioxidant activity in terms of its dependence on the antioxidant concentration (IIC) rather than to relate it to a single antioxidant concentration (SIC) since antioxidant interactions are strongly concentration-dependent and single-point analysis (SIC) cannot be representative for other concentrations. Moreover, the SIC largely eliminates experimental errors of single measurements [32,33]. The slopes of the dependences of the lag time and t_1/2_ on the concentrations of antioxidants studied decreased with increasing anode peak potential of the antioxidants, while the slopes of the dependences of the extent of fluorescein protection calculated from the AOC and of the maximum slope of fluorescence decay on the antioxidant concentrations were not related to the values of their anodic peak potentials (Figure 2).

The interactions between the antioxidants of red cabbage extract (mainly anthocyanins) and exogenous antioxidants were evaluated using the IIC and SIC for all parameters derived from ORAC measurements. In most cases, statistical evaluation of IIC results provided no premises to claim synergy or antagonism of the interactions except for Trolox (synergy when estimated based on AUC-derived percent protection, lag time, and t_1/2_) and TEMPOL (antagonistic interaction when estimated by IIC based on all other parameters except for the lag time).

The synergy of the interactions of Trolox may be related to its low redox potential allowing for the efficient repair of (i) the fluoresceinyl radical and also (ii) radicals of anthocyanins (E_p,a_ of 0.56 V for cyanidin [34]; various cyanidin derivatives are present in the red cabbage extract [13,14]) and other polyphenols present in the extract.

The regeneration of antioxidants apparently plays an important role in the antioxidant interactions. Soluble antioxidants regenerate antioxidants bound to the insoluble food matrix [35]. Glutathione [36] and dihydrolipoic acid [37] can non-enzymatically regenerate ascorbic acid from dehydroascorbic acid. Trolox can be regenerated from the Trolox radical by Coenzyme Q_0_ [38]. Although generally, phenolics are thought to regenerate tocopherol [39], in some situations, a reverse reaction can occur [40]. It may be relevant to the behavior of Trolox whose redox potential is somewhat (by about 0.02 V) lower than that of α-tocopherol [31]. The “pecking order” is compound-dependent: caffeic acid was reported to regenerate tocopherol from the tocopheryl radical, but the phenoxyl radical of *p*-coumaric acid oxidized α-tocopherol [41]. This was due to the E_p,a_ potential of *p*-coumaric acid being about 0.3 V higher than that of caffeic acid [42]. Catechin (E_p,a_ of 0.47 V at pH 5.2 [43]) was reported to regenerate the malvidin 3-glucoside radical [44]; thus, Trolox, whose E_p,a_ is much lower, can be expected to regenerate anthocyanin radicals formed in the system, even if cyanidin derivatives have lower E_p,a_ than malvidin derivatives [45].

The antagonistic interactions of TEMPOL can be mainly attributed to (i) its high redox potential [46], (ii) possible cross-oxidation of other antioxidant radicals, and (iii) the steric hindrance of the nitroxyl group, preventing its interaction with bulky anthocyanin radicals. TEMPOL, having a high redox potential, can oxidize radicals of other antioxidants formed in reactions with the peroxyl radicals or with the fluoresceinyl radical, preventing their further reactions, while the TEMPOL hydroxylamine formed in this reaction is less reactive than the fluoresceinyl radical due to the steric hindrance. As the reactions occurring in the lag phase contribute to the total effect of ORAC, this behavior of TEMPOL contributes to the antagonism of the interaction with this antioxidant, as estimated by fluorescence protection as well. The antagonistic nature of interaction between TEMPOL and red cabbage antioxidants was also found in our previous study in the ABTS^•^ decolorization assay [12].

The present results allow for the comparison of the utility of various parameters derivable from ORAC measurements for the evaluation of interactions between antioxidants. The AUC-based protection of fluorescein is the most widely used parameter. Antagonistic, additive, and synergistic interactions were found when analyzing interactions between various dietary antioxidants, including phenolic acids [47,48,49,50,51,52] and complex food components such as various fractions of pomegranate juice and grape juice [53,54].

In the present study, the AUC-based estimation of the extent of fluorescence protection, lag time and t_1/2_ indicated a synergistic interaction of Trolox with the antioxidants of the red cabbage extract. It seems, therefore, that t_1/2_, which is easy to determine automatically, can be a useful parameter providing information similar to the lag time, whose determination is more cumbersome and subject to some arbitrariness. However, it may be less sensitive than the lag time, as the slope of the fluorescence decay following the lag time contributes to the t_1/2_ value.

In turn, the maximum rate of fluorescence decay does not seem to convey meaningful information about the interaction of antioxidants. This ill-defined final phase of the ORAC assay showed weakly antagonistic interactions but none statistically different from additivity, except for TEMPOL, whose interaction with the red cabbage extract was clearly antagonistic. A limitation of the latter two parameters is the lack of their linear dependence of the antioxidant concentration in the higher concentration range (Figure 1).

The SIC values showed a higher variability with respect to those of the IIC and in some cases, they indicated a different type of interaction than the IIC. It was difficult to classify the type of interactions of ascorbic acid, GSH and Trolox with the extract based on the extent of fluorescence protection and of gallic acid and TEMPOL with the extract based on the lag time measurements using the SIC (Table 5).

Redox properties of antioxidants and their interactions with other antioxidant compounds are crucial for understanding and predicting their behavior in the cell or food matrix. Although the redox potential of an antioxidant does not predict the efficiency of its action, which may depend on kinetic parameters [55], it is a thermodynamic criterion of the possibility of occurrence and direction of reactions with other compounds [31]. As interactions between antioxidants are concentration-dependent, the use of the IIC to consider this dependence seems more appropriate to characterize these interactions than measurements at single concentrations of antioxidants. Further studies of such interactions are of value since revealing synergistic interactions may help reduce the amount of antioxidant supplements necessary to protect food products against oxidation.

## 4. Materials and Methods

### 4.1. Reagents, Materials and Equipment

The Folin–Ciocalteu phenol reagent (cat. no. F9252), 37% hydrochloric acid (CAS no. 7647-01-0, cat. no. 320331), gallic acid monohydrate (CAS no. 5995-86-8; cat. no. 398225), sodium ascorbate (CAS no. 134-03-2, cat. no. 11140), Trolox (CAS no. 53188-07-1, cat. no. 648471), sodium carbonate (CAS no. 497-19-8, cat. no. 106392), ascorbic acid (CAS no. 50-81-7, cat.no. A7506), glutathione, (CAS no. 70-18-8; cat. no. Y0000517), TEMPOL (CAS no. 2226-96-2, cat. no. 176141), and fluorescein sodium salt (CAS no. 518-47-8, cat. no. 46960) were provided by Merck (Poznan, Poland). Sodium dihydrogen phosphate (CAS no. 10049-21-5, cat. no. PM306.500; purity 98–103%) and sodium hydrogen phosphate (CAS no. 7782-85-6, cat. no. SPD579.1; purity 98–102%) produced by BioShop Canada Inc. (Burlington, ON, Canada) were purchased from Lab Empire (Rzeszow, Poland). Glacial acetic acid (CAS no. 64-19-7; cat. no. JT9522-2) and sodium acetate anhydrous (CAS no. 127-09-3; cat. no. BN60/6191; purity ≥ 99%) were obtained from Avantor Performance Materials (Gliwice, Poland). Sodium hydroxide (CAS no. 1310-73-2) was obtained from Warchem (Zakręt, Poland). Red cabbage (*Brassica oleracea* var. *capitata* f. *rubra*), grown in the continental agroclimatic zone, was purchased in a local supermarket in Rzeszów.

Distilled water was purified using a Milli-Q system (Millipore, Bedford, MA, USA). Transparent flat-bottom 96-well plates (cat. no. 655101) (Greiner, Kremsmünster, Austria) were used for the assays. Fluorimetric and absorptiometric measurements were performed in a Spark (Ref. 30086376) multimode microplate reader (Tecan Group Ltd., Männedorf, Switzerland).

### 4.2. Preparation of Red Cabbage Extract

External leaves of the cabbage head were removed and the cabbage (about 50 g) was washed and chopped into about 1 cm × 1 cm × 1 cm fragments; ground in a mortar with 100 mM acetate buffer, pH 5.0 (50 g leaves/450 mL of water); shaken for 30 min; and centrifuged (3000× *g*; 20 min). The supernatant was aliquoted and stored frozen at −80 °C in small portions, thawed only once for no more than 1 month. Such storage conditions did not cause detectable changes in the absorption spectra or an increase in the extent of oxidation as assessed by fluorescence spectra at an excitation wavelength of 460 nm [56]. The composition of the extract was reported in a previous study [12].

### 4.3. Estimation of Anthocyanin Concentration

Anthocyanin concentration was estimated according to a slightly modified procedure of Lee et al. [57]. Briefly, 125 µL aliquots of the extract were added with 875 µL of 0.1 M acetate buffer, pH 4.5, or 1.5 M HCl, and the absorbance of both samples was measured at the absorption maximum (at about 520 nm) and 700 nm.

The anthocyanin concentration c was calculated as

c [µmol/L] = A × dilution × 10^3^)/(ε × l),

where

A = (A_max_ − A_700 nm_)_pH 1_ − (A_max_ − A_700 nm_)_pH 4.5_,

where A_max_ is maximal absorbance; ε is the molar absorption coefficient for kuromarin (ε = 26.9 mM^−1^ cm^−1^); and l is the length of the optical path [cm].

### 4.4. ORAC Assay

Florescence of the solutions containing fluorescein (final concentration 200 nM), AAPH (final concentration 100 mM), red cabbage extract (0–2.75 µM in anthocyanins) and antioxidants (0–5 µM or 0–7.5 µM) in 100 mM sodium phosphate buffer, pH 7.0 (final volume of 200 µL), was measured in a Spark plate reader at 37 °C every two minutes for up to 120 min using excitation and emission wavelengths of 478 nm and 520 nm, respectively. The high concentration of AAPH was used to shorten the reaction time and have it completed within 2 h, even with the highest concentrations of antioxidants used.

From the plots of fluorescence vs. time, the following parameters were calculated: (i) area under curve (AUC), i.e., the sum of fluorescence intensities for all measurements; (ii) lag time, i.e., the time to the onset of rapid loss of fluorescence, determined by finding the intersection point of tangents of the initial phase of slow fluorescence decrease and the phase of rapid fluorescence decay; (iii) t_1/2_, i.e., the time taken for the loss of fluorescence to ½ of the initial value; (iv) slope of maximal fluorescence decay calculated as the decrease in absorbance between two readings preceding fluorescence loss to 50% of the initial value and two readings following it.

From the AUC, the extent of protection of fluorescein fluorescence was calculated as follows:

Protection [%] = 100% × [(AUC for given sample containing fluorescein, AAPH and antioxidant(s)) − (AUC for a sample containing only fluorescein and AAPH)]/[(AUC for sample containing only fluorescein) − (AUC for a sample containing only fluorescein and AAPH)]

### 4.5. Statistics

The experiments were done in triplicate for each concentration range of the antioxidants. The results are presented as arithmetic means ± SD. Statistical significance of differences in the SIC and IIC from the value of 1 (indicating additive interaction) was estimated using the one-tailed Student *t*-test after verification of the normality of the distribution using the Shapiro–Wilk test. Statistical significance of differences between independent sets of experiments was evaluated with the two-tailed Student *t*-test.

## Figures and Tables

**Table 1:** Integrated interaction coefficients (IICs) for the interaction of selected antioxidants with the red cabbage extract derived from the concentration dependence of the extent of fluorescence protection estimated from the AUC. Mean values ± SD; *n* = 3.

System	Concentration Range [µM]	IIC
Extract/ascorbic acid	0.23–2.75/0.42–5.00	1.14 ± 0.09
Extract/ascorbic acid	0.23–2.75/0.63–7.50	1.12 ± 0.11
Extract/gallic acid	0.23–2.75/0.42–5.00	1.06 ± 0.08
Extract/gallic acid	0.23–2.75/0.63–7.50	1.05 ± 0.05
Extract/GSH	0.23–2.75/0.42–5.00	1.20 ± 0.13
Extract/GSH	0.23–2.75/0.63–7.50	1.08 ± 0.11
Extract/Trolox	0.23–2.75/0.42–5.00	1.17 ± 0.08 *
Extract/Trolox	0.23–2.75/0.63–7.50	1.24 ± 0.11 *
Extract/TEMPOL	0.23–2.75/0.42–5.00	0.58 ± 0.18 *
Extract/TEMPOL	0.23–2.75/0.63–7.50	0.87 ± 0.06 *

* *p* < 0.05 with respect to the value of 1 (one-tailed Student’s *t*-test). Green values indicate synergistic interaction and red values indicate antagonistic interaction.

**Table 2:** IIC for the interaction of selected antioxidants with the red cabbage extract derived from the concentration dependence of the lag time of the reaction. Mean values ± SD; *n* = 3.

System	Concentration Range [µM]	IIC
Extract/ascorbic acid	0.23–2.75/0.42–5.00	1.09 ± 0.13
Extract/ascorbic acid	0.23–2.75/0.63–7.50	1.11 ± 0.10
Extract/gallic acid	0.23–2.75/0.42–5.00	1.06 ± 0.07
Extract/gallic acid	0.23–2.75/0.63–7.50	1.12 ± 0.15
Extract/GSH	0.23–2.75/0.42–5.00	1.09 ± 0.12
Extract/GSH	0.23–2.75/0.63–7.50	0.96 ± 0.08
Extract/Trolox	0.23–2.75/0.42–5.00	1.15 ± 0.07 *
Extract/Trolox	0.23–2.75/0.63–7.50	1.22 ± 0.10 *
Extract/TEMPOL	0.23–2.75/0.42–5.00	0.90 ± 0.14
Extract/TEMPOL	0.23–2.75/0.63–7.50	0.95 ± 0.09

* *p* < 0.05 with respect to the value of 1 (one-tailed Student’s *t*-test). Green values indicate synergistic interaction.

**Table 3:** IIC for the interaction of selected antioxidants with the red cabbage extract derived from the concentration dependence of the t_1/2_ of the reaction. Mean values ± SD; *n* = 3.

System	Concentration Range [µM]	IIC
Extract/ascorbic acid	0.23–2.75/0.42–5.00	1.09 ± 0.06
Extract/ascorbic acid	0.23–2.75/0.63–7.50	1.12 ± 0.11
Extract/gallic acid	0.23–2.75/0.42–5.00	1.21 ± 0.13
Extract/gallic acid	0.23–2.75/0.63–7.50	1.12 ± 0.09
Extract/GSH	0.23–2.75/0.42–5.00	1.18 ± 0.15
Extract/GSH	0.23–2.75/0.63–7.50	1.12 ± 0.13
Extract/Trolox	0.23–2.75/0.42–5.00	1.15 ± 0.07 *
Extract/Trolox	0.23–2.75/0.63–7.50	1.21 ± 0.10 *
Extract/TEMPOL	0.23–2.75/0.42–5.00	0.75 ± 0.11 *
Extract/TEMPOL	0.23–2.75/0.63–7.50	0.77 ± 0.09 *

* *p* < 0.05 with respect to the value of 1 (one-tailed Student’s *t*-test). Green values indicate synergistic interaction and red values indicate antagonistic interaction.

**Table 4:** IIC for the interaction of selected antioxidants with the red cabbage extract derived from the concentration dependence of the maximal slope of the fluorescence decay. Mean values ± SD; *n* = 3.

System	Concentration Range [µM]	IIC
Extract/ascorbic acid	0.23–2.75/0.42–5.00	0.96 ± 0.03
Extract/ascorbic acid	0.23–2.75/0.63–7.50	0.93 ± 0.08
Extract/gallic acid	0.23–2.75/0.42–5.00	0.92 ± 0.06
Extract/gallic acid	0.23–2.75/0.63–7.50	0.86 ± 0.12
Extract/GSH	0.23–2.75/0.42–5.00	0.94 ± 0.05
Extract/GSH	0.23–2.75/0.63–7.50	0.87 ± 0.16
Extract/Trolox	0.23–2.75/0.42–5.00	0.86 ± 0.15
Extract/Trolox	0.23–2.75/0.63–7.50	0.96 ± 0.06
Extract/TEMPOL	0.23–2.75/0.42–5.00	0.57 ± 0.11 **
Extract/TEMPOL	0.23–2.75/0.63–7.50	0.76 ± 0.17 *

* *p* < 0.05 and ** *p* < 0.01 with respect to the value of 1 (one-tailed Student’s *t*-test). Red values indicate antagonistic interaction.

**Table 5:** SIC values averaged over the whole concentration range of antioxidants for the interaction of selected antioxidants with the red cabbage extract, estimated by analysis of various parameters derived from the ORAC data. Mean values ± SD; *n* = 3.

System	Concentration Range [µM]	Average SIC			
		Fluorescence Protection	Lag Time	t_1/2_	Maximal Rate
Extract/ascorbic acid	0.23–2.75/0.42–5.00	1.01 ± 0.07	0.88 ± 0.15 *	0.79 ± 0.13 **	0.33 ± 0.10 ***
Extract/ascorbic acid	0.23–2.75/0.63–7.5	1.14 ± 0.08 **	0.93 ± 0.04 **	0.82 ± 0.12 **	0.31 ± 0.11 ***
Extract/gallic acid	0.23–2.75/0.42–5.00	1.11 ± 0.09 **	1.69 ± 0.15 ***	0.83 ± 0.13 **	0.30 ± 0.10 ***
Extract/gallic acid	0.23–2.75/0.63–7.5	1.07 ± 0.03 ***	0.99 ± 0.04 ^a^	0.81 ± 0.11 **	0.30 ± 0.10 ***
Extract/GSH	0.23–2.75/0.42–5.00	1.07 ± 0.17	0.88 ± 0.12 *	0.78 ± 0.13 **	0.35 ± 0.10 ***
Extract/GSH	0.23–2.75/0.63–7.5	1.10 ± 0.07 **	0.96 ± 0.11	0.85 ± 0.11 **	0.30 ± 0.08 ***
Extract/Trolox	0.23–2.75/0.42–5.00	1.03 ± 0.14	1.14 ± 0.12 *	0.75 ± 0.12 ***	0.37 ± 0.08 ***
Extract/Trolox	0.23–2.75/0.63–7.5	1.36 ± 0.35 *	1.19 ± 0.11 **	0.86 ± 0.12 *	0.26 ± 0.11 ***
Extract/TEMPOL	0.23–2.75/0.42–5.00	0.64 ± 0.05 ***	0.96 ± 0.17	0.62 ± 0.05 ***	0.33 ± 0.13 ***
Extract/TEMPOL	0.23–2.75/0.63–7.5	0.72 ± 0.10 ***	0.88 ± 0.16 *	0.58 ± 0.06 ***	0.34 ± 0.14 ***

* *p* < 0.05, ** *p* < 0.01, and *** *p* < 0.001 with respect to the value of 1; ^a^ *p* < 0.01 (with respect to the lower concentration range) (one-tailed Student’s *t*-test). Green values indicate synergistic interaction and red values indicate antagonistic interaction.

## Data Availability

The raw data supporting the conclusions of this article will be made available by the authors on request.

## Supplementary Materials

The following supporting information can be downloaded at: https://www.mdpi.com/article/10.3390/ijms27041859/s1.

## Abbreviations

The following abbreviations are used in this manuscript:

AAPH	2,2′-azobis(2-amidinopropane) dihydrochloride
AUC	area under curve
E_p,a_	anodic peak potential
GSH	glutathione
IIC	integrated interaction coefficient
ORAC	oxygen radical absorbing capacity
SIC	sample interaction coefficient
TEMPOL	4-hydroxy-2,2,6,6-tetramethylpiperidin-1-oxyl

## References

[1] Munteanu I.G., Apetrei C. (2021). Analytical methods used in determining antioxidant activity: A review. Int. J. Mol. Sci..

[2] Kotha R.R., Tareq F.S., Yildiz E., Luthria D.L. (2022). Oxidative stress and antioxidants—A critical review on in vitro antioxidant assays. Antioxidants.

[3] Chen X., Li H., Zhang B., Deng Z. (2022). The synergistic and antagonistic antioxidant interactions of dietary phytochemical combinations. Crit. Rev. Food Sci. Nutr..

[4] Ordoudi S.A., Tsimidou M.Z. (2006). Crocin bleaching assay step by step: Observations and suggestions for an alternative validated protocol. J. Agric. Food Chem..

[5] Prior R.L. (2015). Oxygen radical absorbance capacity (ORAC): New horizons in relating dietary antioxidants/bioactives and health benefits. J. Funct. Foods.

[6] Prieto M.A., Vázquez J.A., Murado M.A. (2015). Crocin bleaching antioxidant assay revisited: Application to microplate to analyse antioxidant and pro-oxidant activities. Food Chem..

[7] Schaich K.M., Tian X., Xie J. (2015). Reprint of “Hurdles and pitfalls in measuring antioxidant efficacy: A critical evaluation of ABTS, DPPH, and ORAC assays”. J. Funct. Foods.

[8] Abeyrathne E.D.N.S., Nam K., Ahn D.U. (2021). Analytical methods for lipid oxidation and antioxidant capacity in food systems. Antioxidants.

[9] Ou B., Chang T., Huang D., Prior R.L. (2013). Determination of total antioxidant capacity by oxygen radical absorbance capacity (ORAC) using fluorescein as the fluorescence probe. J. AOAC Int..

[10] Bisby R.H., Brooke R., Navaratnam S. (2008). Effect of antioxidant oxidation potential in the oxygen radical absorption capacity (ORAC) assay. Food Chem..

[11] Asma U., Bertotti M.L., Zamai S., Arnold M., Amorati R., Scampicchio M. (2024). A kinetic approach to oxygen radical absorbance capacity (ORAC): Restoring order to the antioxidant activity of hydroxycinnamic acids and fruit juices. Antioxidants.

[12] Kut K., Sitarz O., Kapusta I., Bartosz G., Sadowska-Bartosz I. (2025). Interaction of red cabbage extract with exogenous antioxidants. Int. J. Mol. Sci..

[13] Wiczkowski W., Szawara-Nowak D., Topolska J. (2013). Red cabbage anthocyanins: Profile, isolation, identification, and antioxidant activity. Food Res. Int..

[14] Ghareaghajlou N., Hallaj-Nezhadi S., Ghasempour Z. (2021). Red cabbage anthocyanins: Stability, extraction, biological activities and applications in food systems. Food Chem..

[15] Xu K., Peng R., Zou Y., Jiang X., Sun Q., Song C. (2022). Vitamin C intake and multiple health outcomes: An umbrella review of systematic reviews and meta-analyses. Int. J. Food Sci. Nutr..

[16] Dresen E., Lee Z.Y., Hill A., Notz Q., Patel J.J., Stoppe C. (2023). History of scurvy and use of vitamin C in critical illness: A narrative review. Nutr. Clin. Pract..

[17] Wianowska D., Olszowy-Tomczyk M. (2023). A concise profile of gallic acid—From its natural sources through biological properties and chemical methods of determination. Molecules.

[18] Hadidi M., Liñán-Atero R., Tarahi M., Christodoulou M.C., Aghababaei F. (2024). The potential health benefits of gallic acid: Therapeutic and food applications. Antioxidants.

[19] Cassier-Chauvat C., Marceau F., Farci S., Ouchane S., Chauvat F. (2023). The glutathione system: A journey from cyanobacteria to higher eukaryotes. Antioxidants.

[20] Chai Y.C., Mieyal J.J. (2023). Glutathione and glutaredoxin—Key players in cellular redox homeostasis and signaling. Antioxidants.

[21] Theodosis-Nobelos P., Papagiouvannis G., Rekka E.A. (2021). A review on vitamin E natural analogues and on the design of synthetic vitamin E derivatives as cytoprotective agents. Mini Rev. Med. Chem..

[22] Hwang S.J., Lee J.H. (2023). Comparison of antioxidant activities expressed as equivalents of standard antioxidant. Food Sci. Technol..

[23] Tiwari A., Tiwari V., Banik B.K., Sahoo B.M. (2023). Mechanistic role of Tempol: Synthesis, catalysed reactions and therapeutic potential. Med. Chem..

[24] Beigrezaei S., Nasri H. (2017). Tempol as an antioxidant; an updated review on current knowledge. Ann. Res. Antioxid..

[25] Queiroz N.L., Nascimento J.A., Nascimento M.L., Nascimento V.B., Oliveira S.C.B. (2017). Oxidation mechanism of fluorescein at glassy carbon electrode. Electroanalysis.

[26] Selaković M., Aleksić M.M., Kotur-Stevuljević J., Rupar J., Ivković B. (2023). Electrochemical characterisation and confirmation of antioxidative properties of ivermectin in biological medium. Molecules.

[27] Kaimal R., Vinoth V., Salunke A.S., Valdés H., Mangalaraja R.V., Aljafari B., Anandan S. (2022). Highly sensitive and selective detection of glutathione using ultrasonic aided synthesis of graphene quantum dots embedded over amine-functionalized silica nanoparticles. Ultrason. Sonochem..

[28] Yang Z., Zhang D., Long H., Liu Y. (2008). Electrochemical behavior of gallic acid interaction with DNA and detection of damage to DNA. J. Electroanal. Chem..

[29] Pisoschi A.M., Danet A.F., Kalinowski S. (2008). Ascorbic acid determination in commercial fruit juice samples by cyclic voltammetry. J. Anal. Meth. Chem..

[30] Nam D.H., Choi K.S. (2019). Tandem desalination/salination strategies enabling the use of redox couples for efficient and sustainable electrochemical desalination. ACS Appl. Mater. Interfaces.

[31] Buettner G.R. (1993). The pecking order of free radicals and antioxidants: Lipid peroxidation, α-tocopherol, and ascorbate. Arch. Biochem. Biophys..

[32] Kut K., Cieniek B., Stefaniuk I., Bartosz G., Sadowska-Bartosz I. (2022). A modification of the ABTS^•^ decolorization method and an insight into its mechanism. Processes.

[33] Furdak P., Bartosz G., Sadowska-Bartosz I. (2025). Effect of thermal treatment on the antiproliferative and antioxidant activities of garlic. Food Sci. Nutr..

[34] de Lima A.A., Sussuchi E.M., De Giovani W.F. (2007). Electrochemical and antioxidant properties of anthocyanins and anthocyanidins. Croat. Chem. Acta.

[35] Cömert E.D., Gökmen V. (2017). Antioxidants bound to an insoluble food matrix: Their analysis, regeneration behavior, and physiological importance. Compr. Rev. Food Sci. Food Saf..

[36] Winkler B.S. (1992). Unequivocal evidence in support of the nonenzymatic redox coupling between glutathione/glutathione disulfide and ascorbic acid/dehydroascorbic acid. Biochim. Biophys. Acta Gen. Subj..

[37] Guo Q., Packer L. (2000). Ascorbate-dependent recycling of the vitamin E homologue Trolox by dihydrolipoate and glutathione in murine skin homogenates. Free Radic. Biol. Med..

[38] Guo Q., Packer L. (1999). ESR studies of ascorbic acid-dependent recycling of the vitamin E homologue Trolox by coenzyme Q_0_ in murine skin homogenates. Redox Rep..

[39] Zhu Q.Y., Huang Y., Chen Z.Y. (2000). Interaction between flavonoids and α-tocopherol in human low density lipoprotein. J. Nutr. Biochem..

[40] Pedrielli P., Skibsted L.H. (2002). Antioxidant synergy and regeneration effect of quercetin,(−)-epicatechin, and (+)-catechin on α-tocopherol in homogeneous solutions of peroxidating methyl linoleate. J. Agric. Food Chem..

[41] Laranjinha J., Vieira O., Madeira V., Almeida L. (1995). Two related phenolic antioxidants with opposite effects on vitamin E content in low density lipoproteins oxidized by ferrylmyoglobin: Consumption vs regeneration. Arch. Biochem. Biophys..

[42] Masek A., Chrzescijanska E., Latos M. (2016). Determination of antioxidant activity of caffeic acid and *p*-coumaric acid by using electrochemical and spectrophotometric assays. Int. J. Electrochem. Sci..

[43] Munteanu I.G., Apetrei C. (2022). Assessment of the antioxidant activity of catechin in nutraceuticals: Comparison between a newly developed electrochemical method and spectrophotometric methods. Int. J. Mol. Sci..

[44] Rossetto M., Vanzani P., Mattivi F., Lunelli M., Scarpa M., Rigo A. (2002). Synergistic antioxidant effect of catechin and malvidin 3-glucoside on free radical-initiated peroxidation of linoleic acid in micelles. Arch. Biochem. Biophys..

[45] Janeiro P., Brett A.M.O. (2007). Redox behavior of anthocyanins present in *Vitis vinifera* L.. Electroanalysis.

[46] Sadowska-Bartosz I., Bartosz G. (2024). The cellular and organismal effects of nitroxides and nitroxide-containing nanoparticles. Int. J. Mol. Sci..

[47] Skroza D., Šimat V., Vrdoljak L., Jolić N., Skelin A., Čagalj M., Frleta R., Generalić Mekinić I. (2022). Investigation of antioxidant synergisms and antagonisms among phenolic acids in the model matrices using FRAP and ORAC methods. Antioxidants.

[48] Taibi M., Elbouzidi A., Haddou M., Baraich A., Ou-Yahia D., Bellaouchi R., Mothana R.A., Al-Yousef H.M., Asehraou A., Addi M. (2024). Evaluation of the interaction between carvacrol and thymol, major compounds of *Ptychotis verticillata* essential oil: Antioxidant, anti-inflammatory and anticancer activities against breast cancer lines. Life.

[49] Freeman B.L., Eggett D.L., Parker T.L. (2010). Synergistic and antagonistic interactions of phenolic compounds found in navel oranges. J. Food Sci..

[50] Huang W.Y., Majumder K., Wu J. (2010). Oxygen radical absorbance capacity of peptides from egg white protein ovotransferrin and their interaction with phytochemicals. Food Chem..

[51] Liu J., Zhang D., Zhu Y., Wang Y., He S., Zhang T. (2018). Enhancing the in vitro Antioxidant Capacities via the interaction of amino acids. Emir. J. Food Agric..

[52] Pozo-Martínez J., Vázquez-Rodríguez S., Olea-Azar C., Moncada-Basualto M. (2022). Evaluation of ORAC methodologies in determination of antioxidant capacity of binary combinations of quercetin and 3-(3, 4, 5-trihydroxybenzoyl) coumarin derivatives. Arab. J. Chem..

[53] Parker T.L., Miller S.A., Myers L.E., Miguez F.E., Engeseth N.J. (2010). Evaluation of synergistic antioxidant potential of complex mixtures using oxygen radical absorbance capacity (ORAC) and electron paramagnetic resonance (EPR). J. Agric. Food Chem..

[54] Bolling B.W., Chen Y.Y., Chen C.O. (2013). Contributions of phenolics and added vitamin C to the antioxidant capacity of pomegranate and grape juices: Synergism and antagonism among constituents. Int. J. Food Sci. Technol..

[55] Furdak P., Kut K., Bartosz G., Sadowska-Bartosz I. (2025). Comparison of various assays of antioxidant activity/capacity: Limited significance of redox potentials of oxidants/indicators. Int. J. Mol. Sci..

[56] Bartosz G., Grzesik-Pietrasiewicz M., Sadowska-Bartosz I. (2020). Fluorescent products of anthocyanidin and anthocyanin oxidation. J. Agric. Food Chem..

[57] Lee J., Durst R.W., Wrolstad R.E., Eisele T., Giusti M.M., Hach J., Hofsommer H., Koswig S., Krueger D.A., Kupina S. (2005). Determination of total monomeric anthocyanin pigment content of fruit juices, beverages, natural colorants, and wines by the pH differential method: Collaborative study. J. AOAC Int..

