# Supplementary material for: Interaction of Red Cabbage Extract with Exogenous Antioxidants in ORAC Assay
Source: Int J Mol Sci. 2026 Feb 15;27(4):1859. doi: 10.3390/ijms27041859 (PMC12940556; doi:10.3390/ijms27041859)
Supplement: Supplementary file 1 [file ijms-27-01859-s001.zip › ijms-4143589-supplementary.pdf]

Interaction of Red Cabbage Extract with Exogenous Antioxidants in the ORAC assay

Oskar Sitarz [1,2], Grzegorz Bartosz [1] and Izabela Sadowska-Bartosz [1,*]

Supplementary Tables

Table S1. Sample Interaction Coefficient (SIC) for the Extent of Fluorescence Protection

Extract anthocyanins [μM]	Anti-oxidant [μM]	Ascorbic acid	Gallic acid	GSH	Trolox	TEMPOL
0.23	0.42	0.91 ± 0.27	1.05 ± 0.19	0.70 ± 0.10*	0.78 ± 0.25	0.70 ± 0.11*
0.46	0.83	1.02 ± 0.37	1.25 ± 0.18	0.96 ± 0.17	0.97 ± 0.13	0.72 ± 0.11*
0.92	1.67	0.98 ± 0.09	1.13 ± 0.12	1.17 ± 0.42	1.00 ± 0.08	0.65 ± 0.09*
1.37	2.50	1.00 ± 0.11	1.13 ± 0.10	1.09 ± 0.22	1.04 ± 0.07	0.61 ± 0.12*
1.83	3.33	1.02 ± 0.08	1.12 ± 0.05*	1.14 ± 0.25	1.21 ± 0.08*	0.60 ± 0.08**
2.29	4.17	1.00 ± 0.06	0.95 ± 0.16	1.11 ± 0.18	1.08 ± 0.07	0.61 ± 0.08*
2.75	5.00	1.15 ± 0.09	1.15 ± 0.11	1.18 ± 0.18	1.15 ± 0.07*	0.61 ± 0.09*
0.23	0.63	1.23 ± 0.45	1.11 ± 0.25	1.24 ± 0.52	2.14 ± 0.10**	0.58 ± 0.28
0.46	1.25	1.06 ± 0.20	1.07 ± 0.14	1.06 ± 0.29	1.24 ± 0.20	0.60 ± 0.16*
0.92	2.50	1.24 ± 0.30	1.12 ± 0.05*	1.02 ± 0.20	1.16 ± 0.23	0.69 ± 0.09*
1.37	3.75	1.05 ± 0.25	1.07 ± 0.10	1.10 ± 0.16	1.17 ± 0.13	0.82 ± 0.08*
1.83	5.00	1.11 ± 0.16	1.02 ± 0.11	1.09 ± 0.19	1.33 ± 0.16	0.81 ± 0.15
2.29	6.25	1.16 ± 0.20	1.07 ± 0.08	1.05 ± 0.09	1.29 ± 0.11*	0.73 ± 0.16
2.75	7.50	1.15 ± 0.17	1.06 ± 0.07	1.11 ± 0.16	1.22 ± 0.06*	0.78 ± 0.11

*$p < 0.05$, **$p < 0.01$, with respect to the value of 1. Green values, synergistic interaction; red values, antagonistic interaction.

Table S2. Sample Interaction Coefficient (SIC) for the Lag Time

Extract anthocyanins [μM]	Anti-oxidant [μM]	Ascorbic acid	Gallic acid	GSH	Trolox	TEMPOL
0.23	0.42	0.73 ± 0.09*	0.69 ± 0.04**	0.64 ± 0.08*	0.92 ± 0.05	0.67 ± 0.05**
0.46	0.83	0.80 ± 0.07*	1.31 ± 0.09*	0.70 ± 0.08*	1.04 ± 0.09	0.82 ± 0.09
0.92	1.67	0.89 ± 0.11	1.50 ± 0.13*	0.92 ± 0.11	1.22 ± 0.07*	1.05 ± 0.14
1.37	2.50	0.84 ± 0.06*	1.75 ± 0.11**	0.94 ± 0.09	1.28 ± 0.10*	0.93 ± 0.08
1.83	3.33	0.89 ± 0.10	2.11 ± 0.17**	0.93 ± 0.10	1.20 ± 0.13	0.97 ± 0.11
2.29	4.17	1.00 ± 0.08	2.25 ± 0.25**	1.00 ± 0.12	1.18 ± 0.09	1.14 ± 0.16
2.75	5.00	0.98 ± 0.04	2.24 ± 0.19**	1.04 ± 0.13	1.15 ± 0.08	1.12 ± 0.09
0.23	0.63	0.75 ± 0.12*	1.00 ± 0.09	0.93 ± 0.11	1.01 ± 0.07	0.56 ± 0.04**
0.46	1.25	1.06 ± 0.09	1.00 ± 0.12	0.96 ± 0.12	1.16 ± 0.11	0.93 ± 0.11
0.92	2.50	0.86 ± 0.07	0.85 ± 0.07*	0.96 ± 0.10	1.20 ± 0.08*	0.82 ± 0.09
1.37	3.75	0.87 ± 0.08	1.05 ± 0.13	0.98 ± 0.07	1.13 ± 0.10	1.03 ± 0.13
1.83	5.00	0.95 ± 0.10	0.98 ± 0.06	1.00 ± 0.12	1.24 ± 0.12	0.95 ± 0.10
2.29	6.25	1.00 ± 0.09	0.97 ± 0.09	0.89 ± 0.09	1.28 ± 0.09*	0.98 ± 0.09
2.75	7.50	1.04 ± 0.11	1.06 ± 0.12	0.99 ± 0.12	1.34 ± 0.11*	0.90 ± 0.12

*$p < 0.05$, **$p < 0.01$, ***$p < 0.001$ with respect to the value of 1. Green values, synergistic interaction; red values, antagonistic interaction.

Table S3. Sample Interaction Coefficient for the $t_{1/2}$

Extract anthocyanins [μM]	Anti-oxidant [μM]	Ascorbic acid	Gallic acid	GSH	Trolox	TEMPOL
0.23	0.42	0.59 ± 0.03 **	0.62 ± 0.03 **	0.56 ± 0.08 **	0.60 ± 0.06 **	0.58 ± 0.03**
0.46	0.83	0.66 ± 0.06 **	0.72 ± 0.06*	0.64 ± 0.05 **	0.68 ± 0.08 *	0.62 ± 0.06 **
0.92	1.67	0.77 ± 0.08 *	0.82 ± 0.08*	0.77 ± 0.08*	0.75 ± 0.07 *	0.64 ± 0.06**
1.37	2.50	0.81 ± 0.07*	1.07 ± 0.26	0.82 ± 0.08*	0.80 ± 0.08*	0.67 ± 0.08*
1.83	3.33	0.84 ± 0.09	0.89 ± 0.06	0.85 ± 0.09	0.93 ± 0.10	0.69 ± 0.07 *
2.29	4.17	0.88 ± 0.08	0.91 ± 0.12	0.88 ± 0.08	0.87 ± 0.12	0.64 ± 0.03**
2.75	5.00	0.95 ± 0.10	0.94 ± 0.07	0.92 ± 0.09	0.90 ± 0.11	0.53 ± 0.05 **
0.23	0.63	0.63 ± 0.04 **	0.63 ± 0.04**	0.70 ± 0.07*	0.72 ± 0.07 *	0.51 ± 0.08 **
0.46	1.25	0.72 ± 0.06*	0.72 ± 0.05 **	0.78 ± 0.06*	0.76 ± 0.12	0.57± 0.06 **
0.92	2.50	0.82 ± 0.08*	0.79 ± 0.07*	0.80 ± 0.07 *	0.78 ± 0.05 *	0.66 ± 0.09*
1.37	3.75	0.85 ± 0.09	0.84 ± 0.08	0.88 ± 0.06	0.87 ± 0.11	0.55 ± 0.05 **
1.83	5.00	0.90 ± 0.08	0.89 ± 0.09	0.95 ± 0.09	1.02 ± 0.21	0.51 ± 0.06**
2.29	6.25	0.91 ± 0.07	0.91 ± 0.07	0.91 ± 0.06	1.03 ± 0.17	0.57 ± 0.08**
2.75	7.50	0.97 ± 0.09	0.93 ± 0.08	0.98 ± 0.07	1.03 ± 0.05	0.65 ± 0.06 **

*$p < 0.05$, **$p < 0.01$, with respect to the value of 1. Green values, synergistic interaction; red values, antagonistic interaction.

Table S4. Sample Interaction Coefficient for the Maximal Slope of Fluorescence Decay

Extract anthocyanins [μM]	Antioxidant [μM]	Ascorbic acid	Gallic acid	GSH	Trolox	TEMPOL
0.23	0.42	0.46 ± 0.02 ***	0.44 ± 0.01 ***	0.46 ± 0.04 **	0.46 ± 0.04 **	0.47 ± 0.04 **
0.46	0.83	0.43 ± 0.01***	0.42 ± 0.02 ***	0.42 ± 0.04 **	0.44 ± 0.05 **	0.43 ± 0.03 ***
0.92	1.67	0.35 ± 0.06**	0.33 ± 0.03 ***	0.37 ± 0.04 **	0.38 ± 0.02 ***	0.41 ± 0.04 **
1.37	2.50	0.31 ± 0.03***	0.21 ± 0.10 **	0.33 ± 0.05 **	0.34 ± 0.03 ***	0.34 ± 0.03 ***
1.83	3.33	0.28 ± 0.03 ***	0.27 ± 0.03 ***	0.29 ± 0.05 **	0.30 ± 0.03***	0.19 ± 0.05 ***
2.29	4.17	0.25 ± 0.04 ***	0.24 ± 0.03 ***	0.25 ± 0.03 ***	0.27 ± 0.05 **	0.14 ± 0.04 ***
2.75	5.00	0.20 ± 0.03***	0.24 ± 0.02 ***	0.23 ± 0.04 ***	0.23 ± 0.03 ***	0.35 ± 0.11 **
0.23	0.63	0.46 ± 0.02***	0.47 ± 0.05 **	0.43 ± 0.03 ***	0.38 ± 0.01 ***	0.53 ± 0.09 **
0.46	1.25	0.41 ± 0.04**	0.40 ± 0.03 ***	0.36 ± 0.03 ***	0.38 ± 0.07 **	0.44 ± 0.04 **
0.92	2.50	0.36 ± 0.04***	0.34 ± 0.03 ***	0.33 ± 0.03***	0.35 ± 0.01 ***	0.24 ± 0.06 **
1.37	3.75	0.32 ± 0.02***	0.28 ± 0.04 ***	0.28 ± 0.03 ***	0.26 ± 0.04 ***	0.28 ± 0.05 **
1.83	5.00	0.24 ± 0.03***	0.24 ± 0.03 ***	0.23 ± 0.04 ***	0.17 ± 0.06 **	0.52 ± 0.06 **
2.29	6.25	0.21 ± 0.02***	0.21 ± 0.02 ***	0.22 ± 0.01 ***	0.16 ± 0.05 ***	0.51 ± 0.11 *
2.75	7.50	0.17 ± 0.02 ***	0.19 ± 0.01 ***	0.19 ±0.01 ***	0.15 ± 0.03 ***	0.38 ± 0.09 **

**p* < 0.05, ***p* < 0.01, ****p* < 0.001 with respect to the value of 1; ^{b}p < 0.01 with respect to the other concentration range. Green, synergistic interaction; red, antagonistic interaction.
